# Premature white matter aging in patients with right mesial temporal lobe epilepsy: A machine learning approach based on diffusion MRI data

**DOI:** 10.1016/j.nicl.2019.102033

**Published:** 2019-10-23

**Authors:** Chang-Le Chen, Yao-Chia Shih, Horng-Huei Liou, Yung-Chin Hsu, Fa-Hsuan Lin, Wen-Yih Isaac Tseng

**Affiliations:** a Institute of Medical Device and Imaging, College of Medicine, National Taiwan University, Taipei, Taiwan; b Institute of Biomedical Engineering, National Taiwan University, Taipei, Taiwan; c Department of Neurology, National Taiwan University Hospital and College of Medicine, Taipei, Taiwan; d Graduate Institute of Brain and Mind Sciences, College of Medicine, National Taiwan University, Taipei, Taiwan; e AcroViz Technology Inc., Taipei, Taiwan; f Department of Medical Biophysics, University of Toronto, Toronto, Ontario, Canada; g Department of Medical Imaging, National Taiwan University Hospital and College of Medicine, National Taiwan University, Taipei, Taiwan

**Keywords:** White matter brain age, Machine learning, Diffusion MRI, Diffusion spectrum imaging, Mesial temporal lobe epilepsy

## Abstract

Brain age prediction based on machine learning has been applied to various neurological diseases to discover its clinical values. By this innovative approach, it has been reported that the patients with refractory epilepsy had premature brain aging. Of refractory epilepsy, right and left subtypes of mesial temporal lobe epilepsy (MTLE) are the most common forms and exhibit distinct patterns in white matter alterations. So far, it is unclear whether these two subtypes of MTLE would have difference in white matter aging due to distinct white matter alterations. To address this issue, a machine learning based brain age model using diffusion MRI data was established to investigate biological age of white matter tracts. All diffusion MRI datasets were obtained from the same 3-Tesla MRI scanner. To build the brain age prediction model, diffusion MRI datasets of 300 healthy participants were processed to extract age-relevant diffusion indices from 76 major white matter tracts. The extracted diffusion indices underwent Gaussian process regression to build the prediction model for white matter brain age. The model was validated in an independent testing set (*N* = 40) to ensure no overfitting of the model. The model was then applied to patients with right and left MTLE and matched controls (right MTLE: *N* = 17, left MTLE: *N* = 18, controls: *N* = 37), and predicted age difference (PAD) was obtained by calculating the difference between each individual's predicted brain age and chronological age. The higher PAD score indicated older brain age. The results showed that right MTLE exhibited older predicted brain age than the other two groups (PAD of right MTLE = 10.9 years [*p* < 0.05 against left MTLE; *p* < 0.001 against control]; PAD of left MTLE = 2.2 years [*p* > 0.1 against control]; PAD of controls = 0.82 years). Patients with right and left MTLE showed strong correlations of the PAD scores with age of onset and duration of illness, but both groups showed opposite directions of correlations. In right MTLE, positive correlation of PAD with seizure frequency was found, and the right uncinate fasciculus was the most attributable tract to the increase in PAD. In conclusion, the present study found that patients with right MTLE exhibited premature white matter brain aging and their PAD scores were correlated with seizure frequency. Therefore, PAD is a potentially useful indicator of white matter impairment and disease severity in patients with right MTLE.

## Highlights

• A brain age prediction model was developed based on diffusion MRI data.
• Patients with right MTLE exhibited older brain age than those with left MTLE.
• Predicted age difference (PAD) was correlated with seizure frequency in right MTLE.
• Right uncinate fasciculus had highest contribution to the observed PAD in right MTLE.

## 1 Introduction

Approximately one third of patients with epilepsy fail to benefit from medication for seizure control (Kwan and Brodie, 2000). Previous morphometric magnetic resonance imaging (MRI) studies using T1-weighted imaging have shown brain-wide atrophy in patients with medically refractory epilepsy (Bernhardt et al., 2009; McDonald et al., 2008; Riederer et al., 2008). Pardoe et al. (2017) employed a machine learning model based on whole brain T1-weighted images to estimate predicted brain age as the underlying biological age of the brain. They found a significant difference between predicted age and chronological age in individuals with medically refractory focal epilepsy, but did not find such difference in individuals with newly diagnosed focal epilepsy. Predicted age difference (PAD), defined as the difference between predicted brain age and chronological age, can be considered as the deviation from a normal trajectory of brain structural changes due to aging, representing an excess of aging effect of the brain (Cole et al., 2018). Hence, refractory epilepsy patients with greater PAD are considered to have biologically older brains than their peers.

Mesial temporal lobe epilepsy (MTLE) accompanying with mesial temporal sclerosis (MTS) is the most common form of refractory epilepsy in adults (Bernhardt et al., 2013; Blumcke et al., 2002). Prior structural MRI studies have reported that right MTLE and left MTLE exhibit distinct patterns of alterations in brain structures (Besson et al., 2014; Fang et al., 2015; Pail et al., 2010; Pustina et al., 2015). For instance, Pail et al. used voxel-based morphometry to compare structural differences between right and left MTLE groups (2010). They found extensive gray matter volume reduction beyond the affected mesial temporal regions in right MTLE, but not in left MTLE. White matter fiber tracts have been proposed to play an integral role in forming the epileptic network because axons provide a physical basis for seizure propagation to transmit epileptic activity between brain regions (Gross, 2011). Recent diffusion tensor imaging (DTI) studies have found a pronounced difference in the impaired white matter tracts between right and left MTLE, implying two distinct epileptic networks developed in different subtypes of unilateral MTLE (Besson et al., 2014; Fang et al., 2015; Pustina et al., 2015). Neuropsychological studies have also reported that right MTLE and left MTLE present different brain dysfunctions in memory (Frisk and Milner, 1990; Smith and Milner, 1989), emotion (Hermann et al., 2008), and executive function (Hocking et al., 2013). Mounting evidence of distinct structural and cognitive impairments implies that patients with right and left MTLE may experience different severities of cumulative white matter damages, reflecting a difference in neuropathology between these two subtypes of unilateral MTLE. Given the probable difference in white matter impairments between right and left MTLE, it is possible that predicted brain age may be markedly different between these two subtypes.

Most machine learning approaches for brain age prediction adopt a supervised learning strategy to construct a statistical model which relates the features on brain structure MRI data to the corresponding chronological age labels in a group of healthy individuals. The model is trained to fit the age-related trajectory of brain structural changes across lifespan. The resulting model can then be applied to an unseen brain MRI data to predict the respective individual's brain age (Cole and Franke, 2017b; Cole et al., 2017). Such machine-learning-based framework for brain age prediction allows us to evaluate the status of an individual's brain health; an individual's brain is presumably considered healthy if the predicted brain age falls within the normal variation of the prediction (Franke et al., 2010).

Recently machine learning based brain age prediction has been applied to patients with various neurological diseases, such as Alzheimer's disease (Franke et al., 2010), mild cognitive impairment (Gaser et al., 2013), traumatic brain injury (Cole et al., 2015), and refractory epilepsy (Pardoe et al., 2017), and found apparently older predicted brain age than their chronological age. Therefore, PAD is considered as a potential imaging marker of brain health that can identify brain deterioration and may help improve the detection of neurodegenerative disease in its early stage (Cole and Franke, 2017b; Cole et al., 2017).

To create a brain age prediction model, most studies extract features of gray matter or whole brain structures from T1-weighted images (Cole et al., 2015, 2017; Franke and Gaser, 2012; Franke et al., 2010; Varikuti et al., 2018). However, a recent review suggests that white matter microstructure may be more sensitive to subtle changes during the process of aging than gray matter (Liu et al., 2017). For example, cross-sectional studies using DTI have demonstrated clear temporal trajectory of white matter microstructural changes from childhood to late adulthood (Kochunov et al., 2012; Westlye et al., 2010). In particular Westlye et al. (2010) found that the timing of axonal maturation estimated by DTI indices is much earlier than the peak of white matter development estimated by volumetric measurements. Several DTI studies also reported that microstructural changes in white matter fiber tracts are associated with aging-related diseases (Amlien and Fjell, 2014; Teipel et al., 2014; Zhang et al., 2009). Despite potential values of white matter microstructures, only a few studies attempted to extract white matter features from diffusion MRI data to predict an individual's brain age. Mwangi et al. (2013) was the first one to use diffusion scalar indices for white matter brain age prediction. They proved that diffusion indices were valid metrics to project white matter changes during normal aging. Given the well-known role of white matter in lifespan perspective, white matter brain age prediction based on exclusively diffusion imaging is clearly necessitated.

To assess white matter brain age associated with distinct white matter impairments in unilateral MTLE, the present study built a machine learning model using white matter features derived from diffusion spectrum imaging (DSI) data (Wedeen et al., 2005). DSI is an advanced diffusion MRI technique with high angular resolution to characterize intravoxel heterogeneities of fiber architectures, enabling us to reconstruct fiber tractography more accurately than DTI (Wedeen et al., 2008). From the DSI datasets, we calculated a wide array of diffusion indices using the mean apparent propagator MRI (MAP-MRI) algorithm (Ozarslan et al., 2013). We performed tract-based automatic analysis (Chen et al., 2015) to sample the MAP-MRI-derived diffusion indices in 76 predefined fiber tracts as feature inputs for machine learning, and built a white matter brain age prediction model from 300 healthy people. We then applied the model to predict white matter brain age in patients with right and left MTLE and quantified aging-like effect on white matter tract integrity in terms of PAD.

Therefore, the aim of the present study is to characterize white matter brain age in patients with right and left MTLE. We examined the difference in PAD among three study groups, namely right MTLE, left MTLE, and sex- and age-matched healthy participants. We hypothesized that PAD of both patient groups would be larger than that of the healthy group, and the right and left MTLE groups would show a difference in PAD, indicating different status of white matter impairment. Correlations of patients’ PAD with clinical variables including duration of illness, age of onset, and seizure frequency were performed. The results could inform the clinical relevance of premature brain aging in MTLE. Finally, patients’ diffusion indices were normalized to the *z-*scores by a normative model built by another DSI datasets of healthy participants (*N* = 524; age: 7–92 years). The results could provide information about the structural underpinning of premature brain aging as observed in MTLE.

## 2 Methods and materials

### 2.1 Participants

To develop a prediction model of white matter brain age, brain images including T1-weighted images and DSI datasets of 300 healthy individuals, obtained from the National Taiwan University Hospital (NTUH) MRI database, were used as the training set. To confirm the accuracy of the age prediction model, another independent set of 40 healthy individuals from the database was used as the testing set. Detailed information about the training and testing sets including demographics and recruitment criteria are described in Supplementary Material S1. All training and testing datasets were anonymized. Informed consent as approved by the Institutional Review Board of NTUH was given by each participant.

To apply the brain age model to the study groups, patients with chronic MTLE including left MTLE (*N* = 18, 10 men, mean age ± standard deviation: 37.4 ± 8.5 years) and right MTLE (*N* = 17, 9 men, 37.9 ± 8.1 years), and age-matched controls (*N* = 37, 17 men, 38.4 ± 8.3 years) were recruited from the Department of Neurology, NTUH. All participants were matched in handedness assessed by the Edinburgh Handedness Inventory (Oldfield, 1971): left MTLE = 89.9 ± 21.1, right MTLE = 86.5 ± 36.4, controls = 91.8 ± 22.5. None of the participants had a previous history of brain surgery or other neurologic or psychiatric disease.

All patients underwent comprehensive clinical assessments based on the current International League Against Epilepsy classification (Berg et al., 2010), including structural MRI examinations (Coste et al., 2002), long-term video-electroencephalography (EEG) monitoring, neuropsychological testing, and careful interviews to confirm left or right temporal seizure onset. All patients were confirmed to accompany with MTS by a board-certified neurologist (H.H.L.) and two neuroradiologists according to the following MRI findings: (1) smaller hippocampal volume on T1-weighted imaging and abnormal signal hyperintensity in the mesial temporal region on T2-weighted imaging on the epileptic side, and (2) absence of other structural abnormalities, such as MTS occurring in bilateral hemispheres, gray matter heterotopia, tumor, and trauma. Besides visual inspection, we performed a Computational Anatomy Toolbox (CAT, http://www.neuro.uni-jena.de/cat/index.html) with LPBA40 human brain atlas based on SPM12 (Wellcome Trust Center for Neuroimaging, London, UK) to obtain hippocampal volume of two hemispheres for each study group. Long-term video-EEG monitoring was used to confirm locations where epileptic seizures originate in the brain. As shown in Table 1, compared with the healthy control group, the left MTLE group had left hippocampal atrophy and the right MTLE group had right hippocampal atrophy; both groups had no significant volume reductions in the contralateral hippocampus. All participants provided written informed consent, and the Institutional Review Board of the NTUH approved the study. The results of EEG recordings were consistent with the epileptic activity side as identified by structural MRI. Thirty patients (right MTLE: *N* = 16, left MTLE: *N* = 14) were diagnosed with drug-resistant epilepsy in accordance with criteria defined by the International League Against Epilepsy (Kwan et al., 2011).

**Table 1:** Demographics of patients with unilateral mesial temporal lobe epilepsy (MTLE) and controls.

Group	LMTLE	RMTLE	Controls
Subject number	18	17	37
Age (yr)	37.4 (8.5)	37.9 (8.1)	38.4 (8.3)
Sex (M/F)	10/8	9/8	17/20
Handedness (%)	89.9 (21.1)	86.5 (36.4)	91.8 (22.5)
Volume of left hippocampus (cm^3^)	3.24 (0.59)	3.65 (0.38)	3.79 (0.24)
Volume of right hippocampus (cm^3^)	3.91 (0.33)	3.06 (0.89)	3.90 (0.26)
Age of onset (year)	14.5 (5.6)	12.5 (6.7)	–
Duration of illness (year)	22.9 (7.7)	25.5 (9.3)	–
Seizure type (CPS/SPS)	17/1	16/1	–
Seizure frequency (per month)	1.56 (1.73)	1.09 (1.31)	–
Number of AED classes	2.39 (1.33)	2.71 (0.69)	
Secondarily generalized seizure (Y/N)	2/16	1/16	–

Values are reported in [mean (standard deviation)] or [counts].

M = male; F = female; CPS = complex partial seizures; SPS = simple partial seizures.

AED: anti-epileptic drug; LMTLE = left MTLE; RMTLE = right MTLE.

### 2.2 MRI acquisition parameters

Microstructural property of white matter was assessed using various diffusion indices derived from DSI data (Jonasson et al., 2005). All images including the training and testing sets and those of patients and controls were acquired on the same 3-Tesla MRI scanner (Tim Trio; Siemens, Erlangen, Germany) with a 32-channel phased-array head coil. To obtain the anatomical references for image registration, high-resolution T1-weighted imaging was performed using a three-dimensional (3 D) magnetization-prepared rapid gradient-echo sequence; repetition time (TR)/echo time (TE) = 2000/3 ms, flip angle = 9°, field of view (FOV) = 256 × 192 × 208 mm^3^, and acquisition matrix = 256 × 192 × 208, resulting in an isotropic spatial resolution of 1 mm^3^. DSI was performed using a pulsed-gradient spin-echo diffusion echo planar imaging sequence with a twice-refocused balanced echo (Reese et al., 2003; Wedeen et al., 2005) with the imaging parameters *b*_max_ = 4000 s/mm^2^, TR/TE = 9600/130 ms, slice thickness = 2.5 mm, acquisition matrix = 80 × 80, FOV = 200 × 200 mm^2^, and in-plane spatial resolution = 2.5 × 2.5 mm^2^. The diffusion-encoding acquisition scheme followed the framework of DSI (Wedeen et al., 2005), for which 102 diffusion-encoding gradients were applied corresponding to the Cartesian grids in the half-sphere of the 3 D diffusion-encoding space (*q*-space) within a radius of 3 units (Kuo et al., 2008). Because the data in *q*-space are real and symmetrical around the origin, the acquired half-sphere data were projected to fill the other half of the sphere. Each MRI scanning, including T1-weighted imaging and DSI, was completed within 20 min.

### 2.3 Image preprocessing

#### 2.3.1 DSI data reconstruction

Before conducting image reconstruction, image quality of DSI datasets was assessed by a quality assurance pipeline; the procedures included the estimation of signal-to-noise ratio and motion-induced signal dropout in diffusion-weighted images and degree of alignment between T1-weighted images and spatial maps of DSI-derived diffusion indices (Supplementary Material S2 for details). All images in the present study passed the criteria of image quality assurance. The diffusion indices derived from DSI were computed by the regularization version of the framework of mean apparent propagator (ReMAP)-MRI (Hsu and Tseng, 2018; Ozarslan et al., 2013). ReMAP-MRI fitted the signal in *q*-space with a series expansion of Hermite basis functions to describe diffusion in any microstructural environment (Avram et al., 2016). The zero-order term in the expansion series contains the diffusion tensor that characterizes the Gaussian displacement distribution. Higher-order terms in the expansion series are the orthogonal corrections to the Gaussian approximation and are useful for reconstructing the average propagator. The values of axial diffusivity (AD), radial diffusivity (RD), and mean diffusivity (MD) in each voxel were determined by calculating the first eigenvalue, mean of the second and third eigenvalues, and mean of the three eigenvalues of the diffusion tensor, respectively (Alexander et al., 2007). Generalized fractional anisotropy (GFA) was quantified as the standard deviation of the orientation distribution function (ODF) divided by the root-mean square of the ODF (Tuch, 2004). Non-Gaussianity (NG) indices including NG, NG parallel (NGP) to, and NG orthogonal (NGO) to the principal eigenvector of the diffusion tensor were estimated by quantifying the dissimilarity between the propagator and its Gaussian part (Ozarslan et al., 2013). In the present study, we used these seven diffusion indices, namely GFA, AD, RD, MD, NG, NGO, and NGP, to represent various aspects of microstructural property of white matter, such as degree of myelination, fiber calibers, fiber density, and fiber damage (Alexander et al., 2011; Falangola et al., 2013; Kumar et al., 2012).

#### 2.3.2 Tract-specific feature extraction

**Fig. 1:**
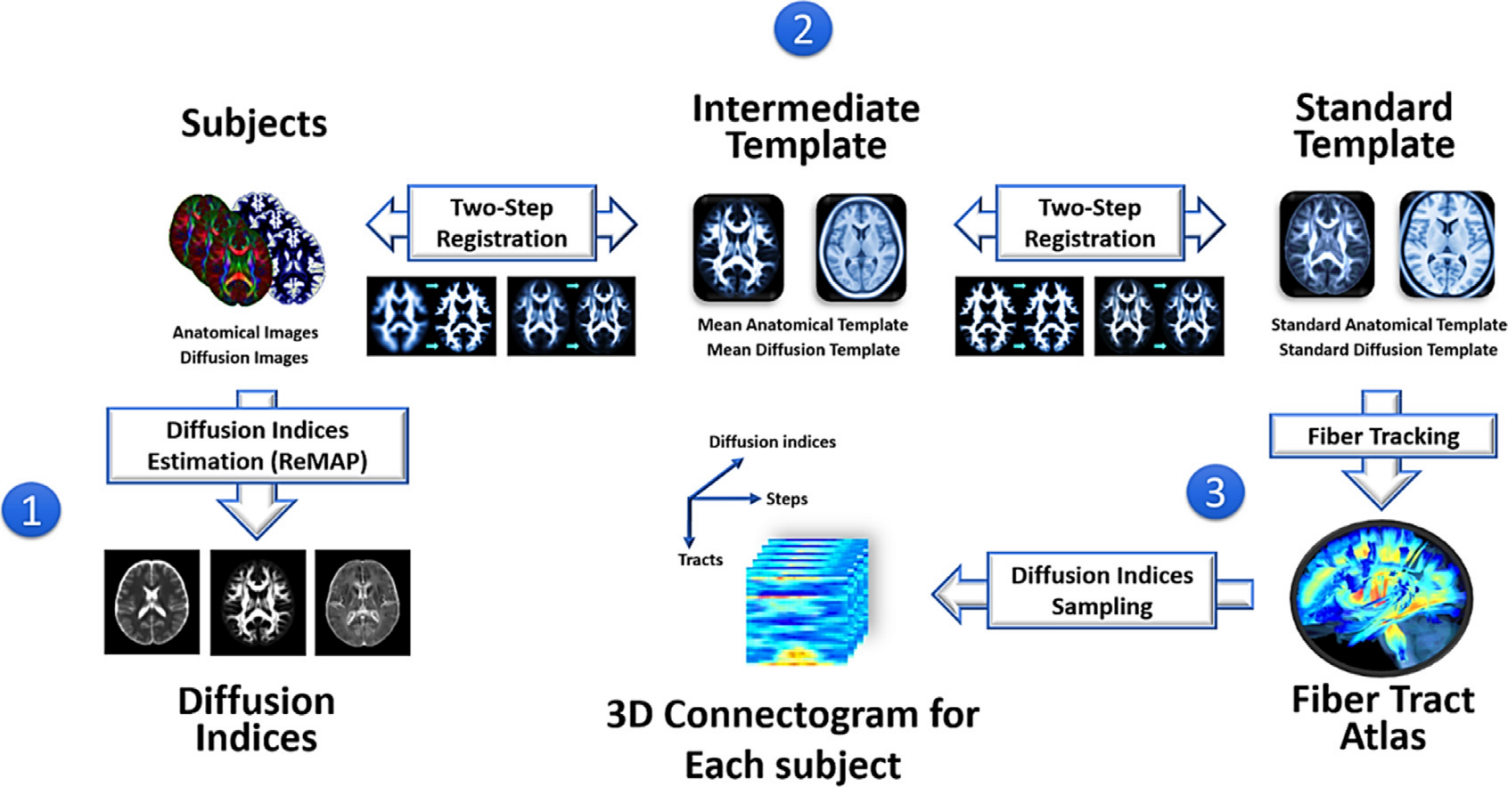
Flow chart of tract-based automatic analysis. (1) The diffusion indices of the microstructural properties of white matter were estimated by the regularized mean apparent propagator method for each diffuse spectrum imaging dataset. (2) The deformation maps of registration were calculated through two-step registration from each individual's native space to standard template space. (3) The predefined coordinates of 76 major white matter tracts were transformed to each individual's native space via the deformation maps to sample the diffusion indices. Finally, the output of tract-based analysis was formatted into a three-dimensional connectogram for each participant (*x*-axis: 100 steps along sampling coordinates; *y*-axis: 76 white matter tract bundles; *z*-axis: 7 diffusion indices).

To extract effective features of white matter tract integrity for machine learning, tract-based automatic analysis was conducted to sample the diffusion indices from 76 predefined major fiber tract bundles over the whole brain (Chen et al., 2015) (Fig. 1). The 76 major fiber tract bundles were built in a DSI template, NTU-DSI-122 (Hsu et al., 2015), using deterministic streamline-based tractography with multiple regions of interest defined in the automated anatomical labeling atlas (Tzourio-Mazoyer et al., 2002). The sampling coordinates of the 76 tracts were transformed from NTU-DSI-122 to individual DSI datasets with corresponding deformation maps. The deformation maps were obtained through two-step registration, which included anatomical information provided by the T1-weighted images (Ashburner and Friston, 2011) and microstructural information provided by DSI datasets (Hsu et al., 2012). The sampling coordinates were aligned with the proceeding direction of each fiber tract bundle, and the values of diffusion indices were sampled in native space along the sampling coordinates that were normalized and divided into 100 steps. Finally, we obtained the output of tract-based analysis for each participant, called a 3 D connectogram (*x*-axis: 100 steps along sampling coordinates; *y*-axis: 76 white matter tract bundles; *z*-axis: 7 diffusion indices). The 3 D connectograms of all the participants, including the training and testing sets, MTLE group, and controls, were used to extract white matter features.

### 2.4 Machine learning prediction of white matter brain age

In the present study, the 3 D connectograms of 300 training datasets were used in the machine learning model to regress age. We developed a prediction model to predict white matter brain age. The model utilized the 76 white matter tract bundles over the whole brain, including bilateral association and projection fibers, and the callosal fibers (Chen et al., 2015) as the features.

To train a model accurately and effectively, the feature preprocess, including smoothing, normalization, and age-based weighted average, was performed before machine learning analysis. First, to reduce local variations along the tract bundles, the diffusion indices along 100 steps of a tract bundle were smoothed by a one-dimensional Gaussian convolution kernel. Second, each step index over subjects was normalized to zero mean and unit variance. Third, the step indices along each tract bundle were calculated into a weighted average. The weight on each step was determined by how strongly the step index was associated with the aging effect. A general linear model was used to fit diffusion indices on each step, with the diffusion index being the dependent variable and the age and age square factors being the independent variables. The *p*-value of the *F* statistic of the general linear model was calculated with minus common logarithm (-*log*_10_) as the weight. Finally, the original 3 D connectograms were reduced to 532 tract features (76 weighted averages × 7 diffusion indices) for each participant.

Because the dimension of the feature space (532 features) was too large in contrast to the current sample size, we used autoencoders (Hinton and Salakhutdinov, 2006) to compress the original features to a lower dimension. The autoencoder was trained by one hidden layer with 179 neurons, which entailed the mean square error with sparsity regularization as the loss function, 1600 training epochs, and optimization by a scaled conjugate gradient-descent algorithm (Møller, 1993). It compressed the 532 original features down to 179 compact features with a 99.55% reconstruction rate. The compact features served as the inputs for the white matter brain age modeling.

**Fig. 2:**
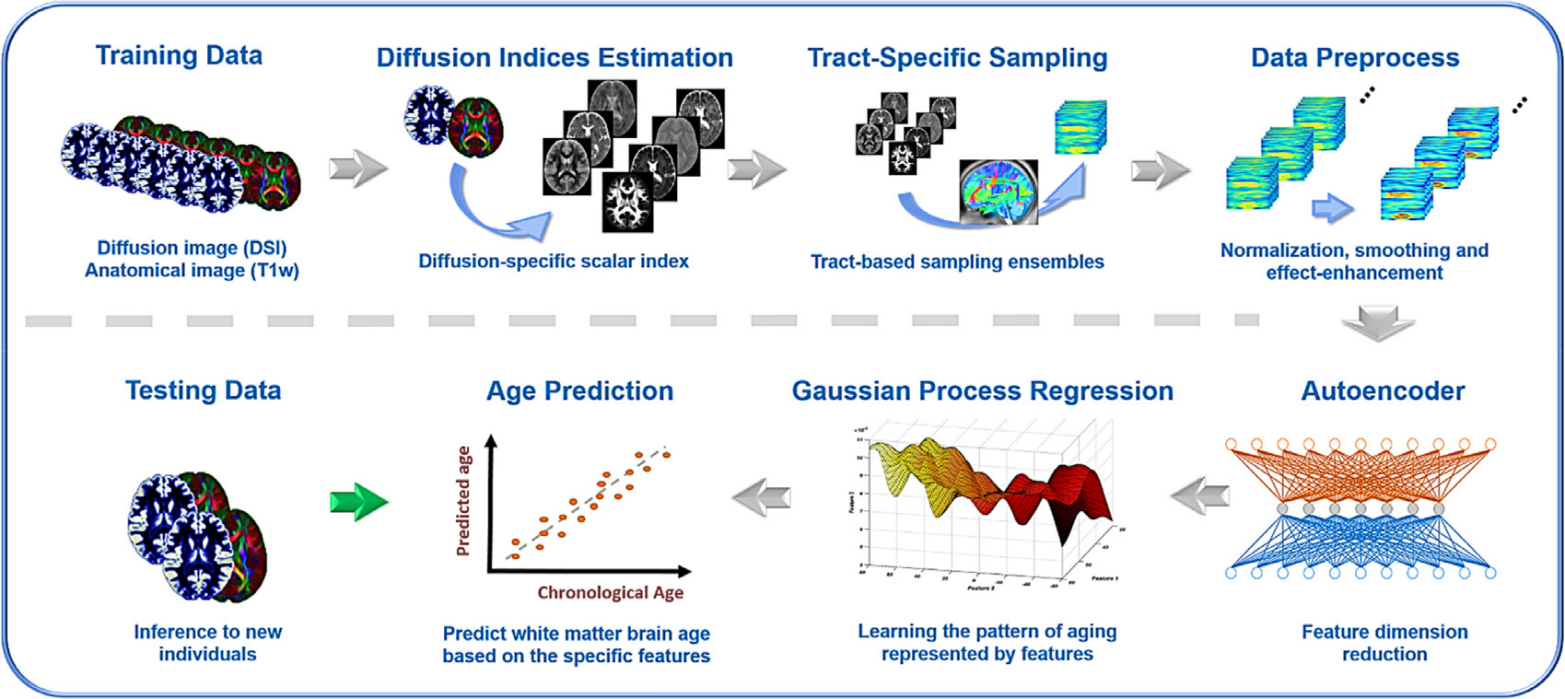
Pipeline of white matter brain age prediction model based on whole brain white matter tracts. The training datasets (*N* = 300) went through the procedures of diffusion index estimation, tract-based automatic analysis, and data preprocessing to generate age-associated features of white matter tracts. These features were compressed by the autoencoder and the compact features were modeled to estimate age with Gaussian process regression. Another independent test dataset (*N* = 40) was used to test the generalizability of the model.

Machine learning analysis was conducted using the Statistics and Machine Learning Toolbox implemented in MATLAB R2018a, and the analysis ran on the compact features (Fig. 2). Gaussian process regression (GPR) model was defined using compact features and sex as the independent variables and age as the dependent variable (Rasmussen, 2004). GPR model is a nonparametric kernel-based probabilistic model. Since aging is a complex process that alters various brain structures at different rates, it is appropriate to regress the age effect through GPR, which amalgamates nonlinear and Gaussian probabilistic properties to estimate continuous variables such as age (Gutierrez Becker et al., 2018).

### 2.5 Model evaluation

Model evaluation was conducted to assess the model's accuracy and to ensure unbiased demonstration of model generalizability between the training and testing sets. Here, we introduced three metrics to quantify model performance, namely Pearson correlation (*r*), root-mean-square error (RMSE), and mean absolute error (MAE) between chronological age and predicted age. A higher correlation indicated better linear consistency of the prediction, and lower RMSE and MAE indicated smaller predicted error between predicted age and chronological age in the normal population. First, the performance of the prediction model derived from the training set was assessed by running a 10-fold cross validation (Fushiki, 2011). One-tenth of the training data were selected randomly to serve as a temporary testing set, and the remaining data were used for temporary model definition. Age was predicted on the temporary testing set by the temporary model iteratively until all the training datasets had been included in the temporary testing set. This validation method provided accurate performance estimation of the training set. Next, the model's generalizability was evaluated by applying the prediction model to the independent testing set (*N* = 40) to predict white matter brain age. Here, the model was built using the entire training set. Having confirmed the model performance, the model was applied to the study groups to characterize white matter brain age in patients with unilateral MTLE.

### 2.6 Brain age characterization for unilateral MTLE

#### 2.6.1 PAD comparison

The PAD scores were obtained by subtracting chronological age from predicted age. The discrepancy between chronological age and predicted brain age could be used as a metric to statistically compare or relate with other measured characteristics of the participants. The PAD scores were statistically compared using analysis of covariance (ANCOVA) to test for group differences among patients with right and left MTLE, and the controls while controlling for age, sex, and handedness as confounders. In the *post hoc*, pairwise comparisons with two-sample *t* tests were carried out to test the between-group differences in the PAD scores and were adjusted for multiple comparisons using Bonferroni correction. The aforementioned statistical analyses were performed using IBM SPSS Statistics version 20.

#### 2.6.2 Regression analysis to evaluate tract contribution

Regression analysis between statistically significant PAD scores and white matter tract alteration was conducted to investigate the structural underpinning of aberrant brain age in patients. To quantify white matter alteration, we introduced a normative model of white matter tract characteristics in healthy participants (*N* = 524; age: 7–92 years). Like the normative model for bone mineral density examination (Zhou et al., 2010), our normative model provided the statistical parameters (i.e. population mean and population standard deviation) of each white matter tract in each diffusion index according to age and sex (Supporting Information S3). To quantify the degree of white matter alteration in patients with MTLE, the *z*-scores of the diffusion indices for each tract bundle were estimated by subtracting the population mean and dividing by the population standard deviation (Sedgwick, 2010). For each patient, diffusion indices of white matter tracts could be transformed into the *z*-scores by comparing them to the age- and sex-matched population. A higher magnitude of *z*-score indicated larger deviation from the normal population. Mass univariate analysis (one-sample *t*-test) was conducted to test the *z*-score of each diffusion index for each tract to identify the tracts that were significantly altered in patients. After multiple comparison adjustments, effect size was calculated for the tracts with significant deviations (Hedges and Olkin, 1984) to assess the quantitative magnitude of group effect in the *z*-scores. The tracts with top 5% of effect size were selected for the following principal component analysis (PCA) (Abdi and Williams, 2010). The first principal component was selected as the representative component, which was the linear combination of the tracts with top 5% of effect size. We used this representative component as the independent variable for the general linear model to explain the variance in the PAD scores.

#### 2.6.3 Correlation analysis of clinical variables

Within the MTLE groups, the PAD scores were assessed for correlation with three clinical variables, namely age of disease onset, duration of illness, and seizure frequency. To avoid the confounding effect, factors of age, sex, and number of anti-epileptic drug classes were regressed out. Nonparametric correlation was adopted when the variables did not follow normal distribution.

## 3 Results

### 3.1 White matter brain age prediction model

**Fig. 3:**
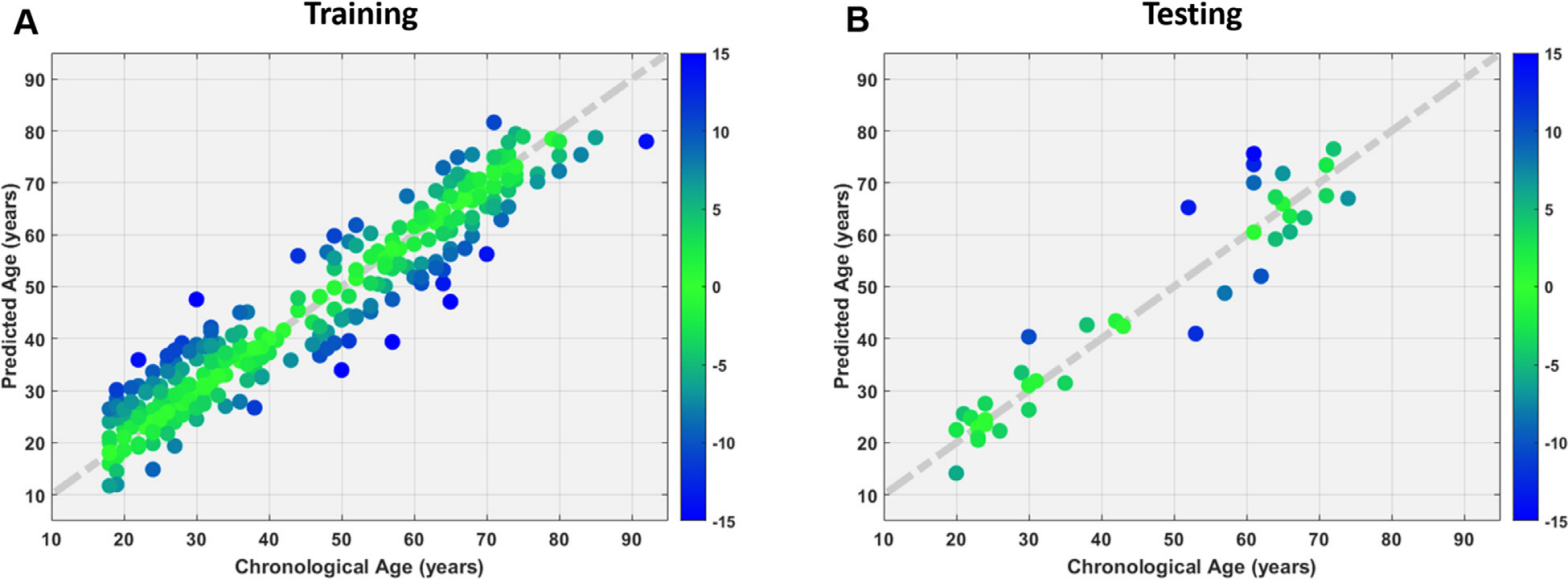
Whole brain based machine learning model showed accurate age prediction in the training (A) and testing (B) sets. Chronological age (*x*-axis) is plotted against predicted age (*y*-axis). The diagonal dashed line represents the line of identity. The color spectrum denotes the absolute error of each individual's predicted age.

The model predicted each individual's age for both the training and testing datasets with satisfactory performance, for the training set (Fig. 3A), Pearson correlation *r* = 0.954, RMSE = 5.78 years, and MAE = 4.61 years, and for the testing set (Fig. 3B), *r* = 0.959, RMSE = 6.50 years, and MAE = 5.08 years. Comparable performance was noted in both training and testing sets, indicating no overfitting problem in the prediction model.

### 3.2 Demographics evaluation

The demographic data were not significantly different among the right MTLE, left MTLE, and control groups in terms of age (*F*(2,69) = 0.081, *p* = 0.922), sex ( χ^2^ = 0.524, *p* = 0.769),and handedness (*F*(2,69) = 0.237, *p* = 0.789). Table 1 summarizes clinical information about seizures in patients with MTLE. Age of disease onset, duration of illness, proportion of seizure types, and seizure frequency were not significantly different between two patient groups (age of onset: *t*(33) = 0.931, *p* = 0.359; duration of illness: *t*(33) = 0.888, *p* = 0.381; seizure type: χ^2^ (1) = 0.002, *p* = 0.967; seizure frequency: *t*(33) = 0.891, *p* = 0.379). In the voxel-based morphometry analysis, the left MTLE group had a significantly smaller left hippocampal volume than the right MTLE group and the controls, whereas the right MTLE group had a significantly smaller right hippocampus than the other groups (left hippocampal volume: *F*(2,69) = 12.3, *p* < 0.001; right hippocampal volume: *F*(2,69) = 18.8, *p* < 0.001; see Table 1).

### 3.3 PAD comparison among right MTLE, left MTLE, and controls

**Fig. 4:**
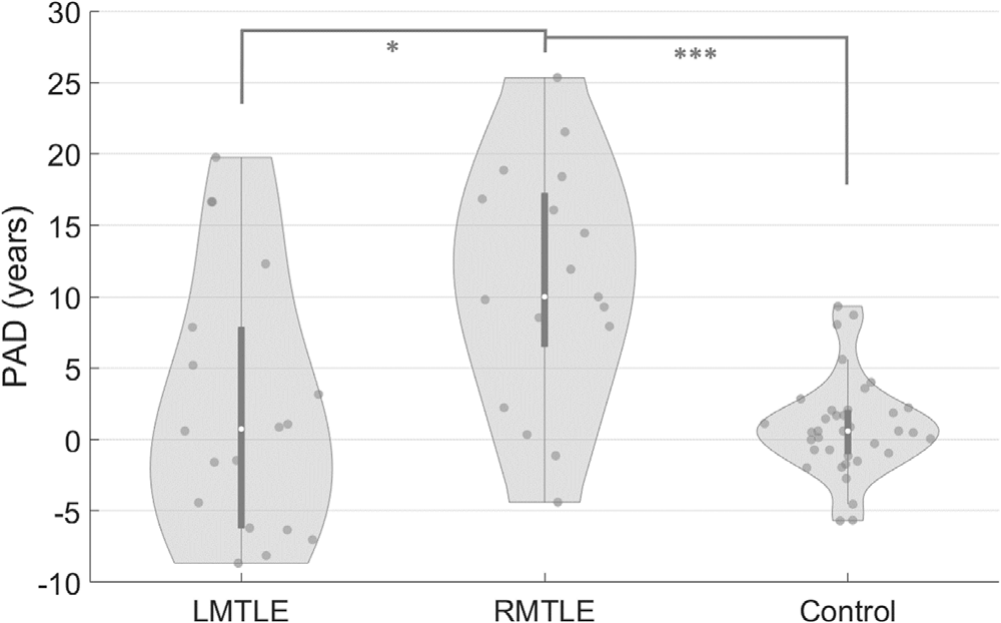
The right mesial temporal lobe epilepsy (RMTLE) group showed significantly increased predicted age difference (PAD) scores compared with the left mesial temporal lobe epilepsy (LMTLE) and control groups. The PAD scores were calculated by subtracting chronological age from predicted white matter brain age for each individual.

The results of ANCOVA showed that there was a significant difference (*F*(2, 66) = 14.578, *p* < 0.001) in the PAD scores among the right MTLE (PAD = 10.94 ± 8.30), left MTLE (PAD = 2.24 ± 9.07), and control (PAD = 0.82 ± 3.36) groups. The *post hoc* pairwise comparison showed that patients with right MTLE had significantly higher PAD scores than those in the left MTLE group (PAD: *t*(33) = 2.956, p* < 0.05; Bonferroni corrected) and controls (PAD: *t*(18.5) = 4.848, p* < 0.001; Bonferroni corrected), whereas no significant difference was found between left MTLE and controls (PAD: *t*(19.3) = 0.641, p* > 0.1; Bonferroni corrected) (Fig. 4).

### 3.4 White matter alterations explained increased PAD in right MTLE

**Fig. 5:**
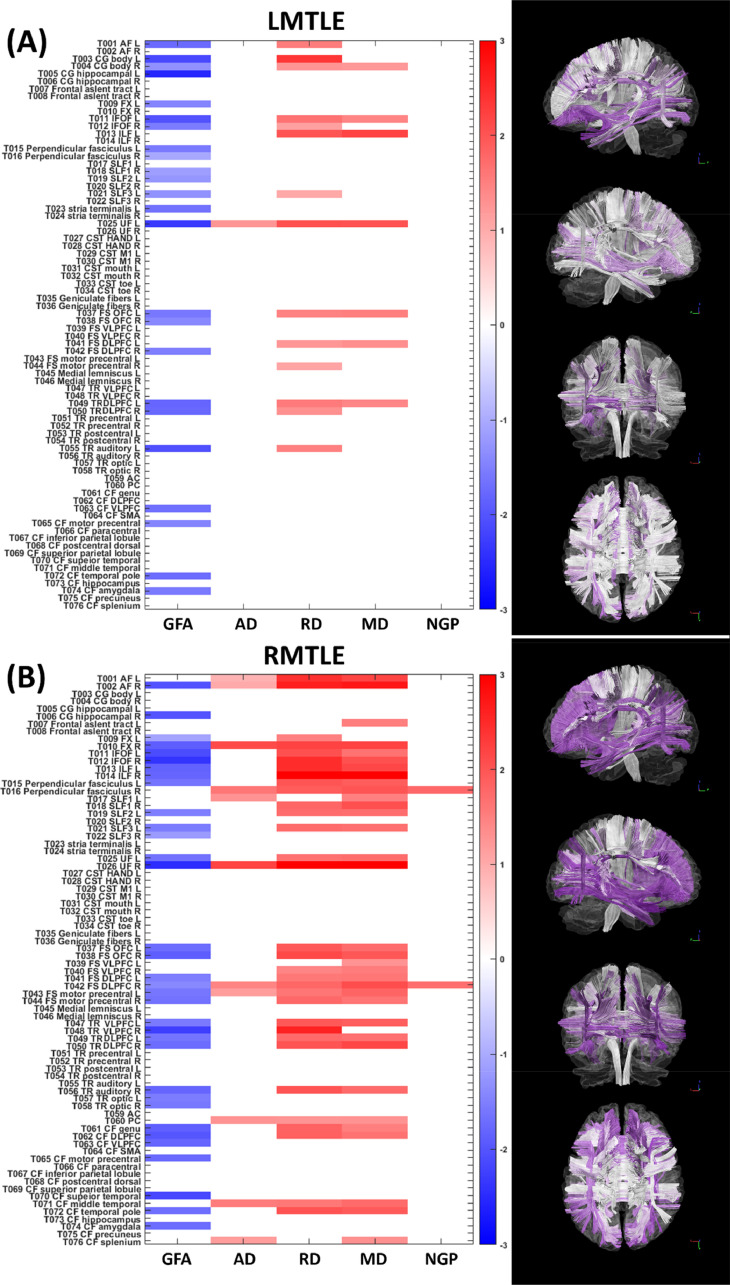
The statistic plots show the white matter tracts with significant differences in the *z*-scores in patients with left mesial temporal lobe epilepsy (LMTLE) and patients with right mesial temporal lobe epilepsy (RMTLE) with respect to the normative model. The shade of the color encodes the magnitude of the *z*-score. The blue and red colors denote negative and positive *z*-scores, respectively. Negative *z*-scores indicate that the diffusion indices in patients were lower than those in normal population, whereas positive *z*-scores indicate higher diffusion indices in patients. The columns from left to right in each statistic plot correspond to five diffusion indices, i.e. generalized fractional anisotropy (GFA), axial diffusivity (AD), radial diffusivity (RD), mean diffusivity (MD), and non-Gaussian parallel (NGP). The right column demonstrates the visualization of the tract bundles encoded with the statistic. The darker purple color indicates the higher magnitude of the total *z*-score that is the summation of the absolute *z*-scores over diffusion indices.

In two patient groups, we used the normative model to transform the diffusion indices into the *z*-scores to indicate the magnitude of deviations compared with the normative population. After testing the *z*-scores for each tract, we identified 46 and 113 tracts in patients with left and right MTLE, respectively, showing significant differences in any of the diffusion indices compared with the corresponding normal population. Fig. 5 shows the significant *z*-score of each tract and each diffusion index from patients with left and right MTLE.

To investigate the underlying tract impairment that leads to the significantly increased PAD scores in patients with right MTLE, we calculated the effect size of each diffusion index for the tracts that showed significant deviations of the *z*-scores in patients with right MTLE. The tracts with effect sizes within top 5% were selected as candidates to explain the overestimated PAD scores. As a result, 27 features were selected to represent the most altered tracts and treated them as the initial variables to explain the variance of PAD.

These 27 features were transformed into principal components through PCA. The first component served as the representative component that explained the largest portion of the variance in the initial variables from right MTLE. Specifically, the first component explained 52.6% of the variance of the initial variables. The first component was used as the independent variable to regress the PAD scores of right MTLE in simple linear regression model. In the regression model, the first component significantly explained the variance of PAD (*F*(1,15) = 13.7, *p* < 0.01; *R*-squared = 0.477). The result indicated that altered white matter tracts significantly explained the variance of the PAD scores. The contributions of the tracts to the first component are displayed in Table 2. The five tracts with the highest weights were the right uncinate fasciculus (22.7% of the weights in the first component), the right frontal striatum of the orbitofrontal cortex (9.7%), the left inferior fronto-occipital fasciculus (9.0%), the left inferior longitudinal fasciculus (8.0%) and the right perpendicular fasciculus (7.6%). Those white matter tracts were the tracts most attributable to the increase in PAD in patients with right MTLE. In addition, we found that RD and MD exhibited higher weights (44.5% for RD; 39.8% for MD) than GFA (10.9%) and AD (4.8%).

**Table 2:** Contributions of the tracts to the first principal component.

Tract	Proportion
Right UF	22.7%
Right FS OFC	9.7%
Left IFOF	9.0%
Left ILF	8.0%
Right perpendicular fasciculus	7.6%
Right TR dorsal part	6.0%
Right IFOF	5.4%
Left perpendicular fasciculus	5.4%
CF genu	4.9%
Right FS motor precentral gyrus	3.8%
Left FS motor precentral gyrus	3.7%
Left FS OFC	3.6%
Right FS DLPFC	3.2%
Left AF	2.9%
Left SLF II	2.4%
Right SLF III	1.8%

AF: arcuate fasciculus; CF: callosal fibers; DLPFC: dorsal lateral prefrontal cortex; FS: frontal-striatum; IFOF: inferior frontal occipital fasciculus; ILF: inferior longitudinal fasciculus; OFC: orbitofrontal cortex; SLF: superior longitudinal fasciculus; TR: thalamic radiation; UF: uncinate fasciculus;.

### 3.5 Correlation between clinical variables and PAD in MTLE patients

There was slight but significant correlation between predicted residuals (i.e., predicted age subtracted by chronological age) and age (*r* = −0.349, *p* < 0.001) in the training set of the prediction model. To remove the variance of confounding factors, a partial correlation approach was used to examine the relationship between PAD and clinical variables, namely age of onset, duration of illness, and seizure frequency. Spearman correlation coefficient was used when the variable did not follow the normality rule. In the right MTLE group, there was a moderate negative correlation between age of onset and PAD (Pearson *r* = −0.511, *p* = 0.036; Fig. 7A), indicating that PAD became larger if disease onset was earlier. By contrast, duration of illness and PAD were positively correlated (Pearson *r* = 0.501, *p* = 0.040; Fig. 7B), indicating that the longer the duration of illness, the higher the PAD score was. Additionally, there was a significantly positive correlation between seizure frequency and PAD (Spearman *r* = 0.635, *p* = 0.007; Fig. 7C), indicating that PAD increased with seizure frequency. In left MTLE, however, the correlation in age of onset and duration of illness against PAD revealed the opposite patterns (left MTLE: age of onset: Spearman *r* = 0.591, *p* = 0.014; duration of illness: Pearson *r* = −0.484, *p* = 0.049; Fig. 7 D&E). In addition, there was no correlation between seizure frequency and PAD in left MTLE (Spearman *r* = −0.152, *p* = 0.559; Fig. 7F). Besides the standard correlation analyses, in order to provide reliable results in the presence of relatively small sample size, we conducted the bootstrap analyses to estimate the empirical distribution of the correlation coefficients. The results of bootstrap analyses were shown in the Supporting Information S4.

## 4 Discussion

The present study developed a multivariate GPR model to predict white matter brain age using diffusion indices of the 76 major fiber tract bundles. The model was employed to predict brain age and the resulting PAD score were used to assess age-related white matter alterations. Patients with right MTLE showed higher white matter brain age than did patients with left MTLE and healthy controls. Right MTLE and left MTLE exhibited opposite relationships of PAD with age of onset and duration of illness. In addition, PAD was positively correlated with seizure frequency in right MTLE. By performing the linear regression analysis for the principal components of the 27 features that best distinguished right MTLE from healthy controls, we identified the right uncinate fasciculus that showed highest contribution to the elevated PAD in patients with right MTLE.

### 4.1 Premature white matter aging in patients with right MTLE

A between-group comparison demonstrated that patients with right MTLE had significantly higher PAD scores than patients with left MTLE and healthy controls (Fig. 4). Subsequent analyses of the *z*-scores of the 76 major fiber tracts in different diffusion indices further showed that patients with right MTLE exhibited more severe and widespread white matter alterations than patients with left MTLE (Fig. 5). Our findings are in line with the findings of recent MRI studies on unilateral MTLE (Besson et al., 2014; Fang et al., 2015; Pail et al., 2010; Pustina et al., 2015). A morphometric study using T1-weighted imaging reported that right MTLE exhibited more extensive gray matter atrophy in the ipsilateral and contralateral temporolimbic systems than did left MTLE (Pail et al., 2010). A DTI study using network-based analysis also found that right MTLE showed more disrupted structural connectivity between brain regions in the bilateral limbic and temporal lobes (Besson et al., 2014). Therefore, our findings suggest that patients with right MTLE undergo more widespread white matter deterioration which might account for the apparent premature white matter aging.

**Fig. 6:**
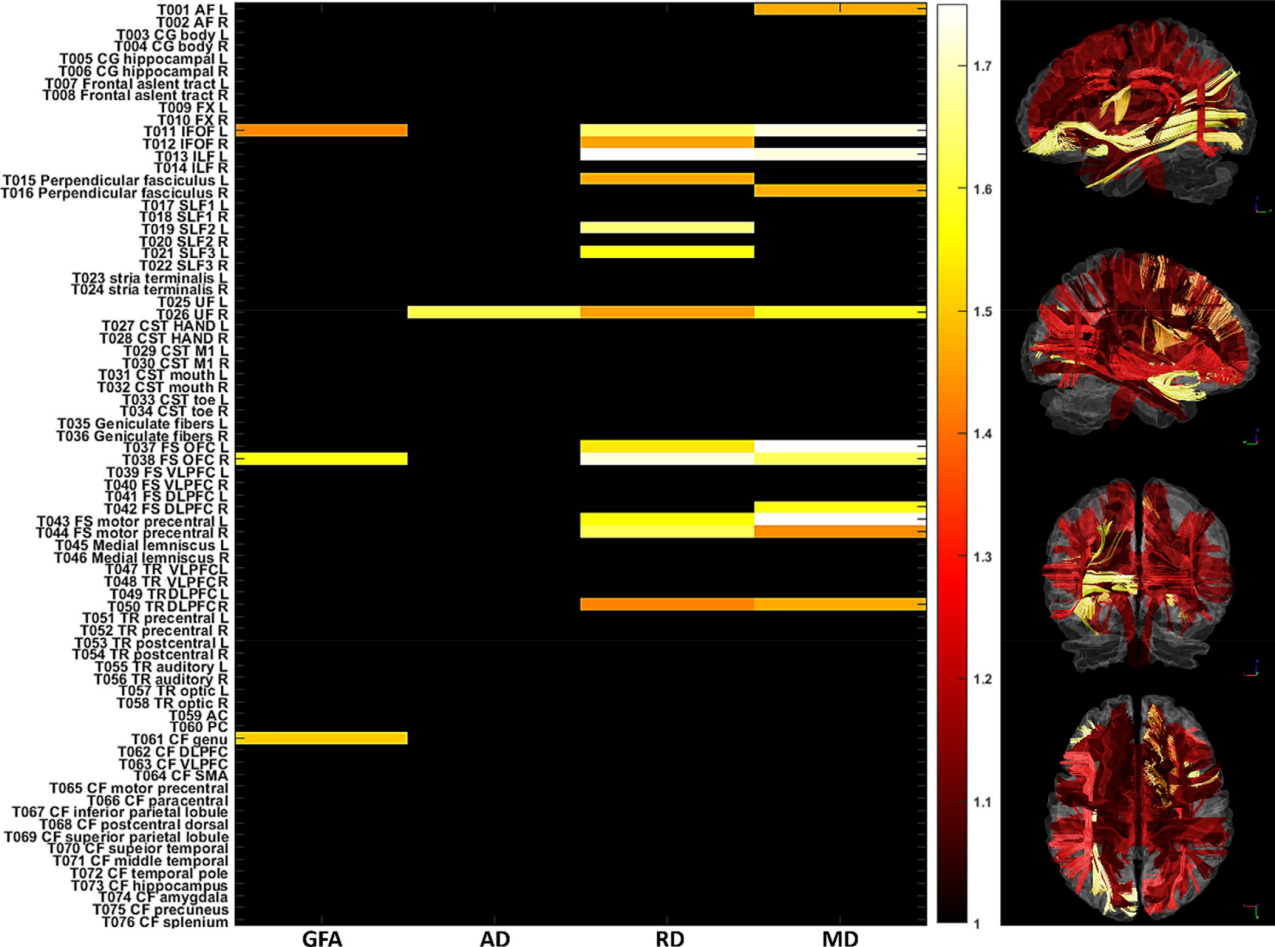
The statistic plots show the white matter tracts whose effect sizes of the *z*-scores were within top 5%. The color spectrum encodes the effect size of the *z*-score. The horizontal and vertical axes indicate the diffusion indices and tracts, respectively. The right column demonstrates the visualization of tract bundles encoded with the effect size of the *z*-scores. The brighter color indicates the higher magnitude of the effect size.

Further analysis in patients with right MTLE using PCA and a general linear model revealed that several white matter tracts and associated diffusion indices were most attributable to the increase in PAD. Most of these white matter tracts belong to association fibers connecting to the temporal lobe (i.e., uncinate fasciculus, inferior fronto-occipital fasciculus, inferior longitudinal fasciculus, superior longitudinal fasciculus) and projection fibers of frontostriatal connection. Among these tracts, the right uncinate fasciculus was the one most contributing to the increase in PAD (Fig. 6 and Table 2). The uncinate fasciculus is a major tract connecting the orbitofrontal lobe with the temporal lobe and a part of the limbic lobe (Kier et al., 2004). Neuroimaging studies have extensively reported structural and functional impairments in the ipsilateral uncinate fasciculus in intractable unilateral MTLE caused by MTS (Chassoux et al., 2004; Diehl et al., 2008; Rodrigo et al., 2007), suggesting a crucial role of the uncinate fasciculus in seizure propagation from the mesial temporal lobe to the frontal lobe (Mayanagi et al., 1996). In addition, Lee et al. (2013) suggested that some tracts with late myelination, such as inferior longitudinal fasciculus and superior longitudinal fasciculus, exhibited more damages than the tracts with early myelination in patients with temporal lobe epilepsy. Such association fibers with late myelination of cortico-cortical circuitry might be affected by excitotoxic effect of seizure propagation (de Lanerolle and Lee, 2005; Lee et al., 2013). Interestingly, we also found that RD, which is usually considered to be related to the degree of myelination (Aung et al., 2013; Basser, 1997), was the most representative diffusion index to describe white matter features that contributed to the increased PAD. In fact, the excitotoxic effect of seizure activity leads to demyelination in epilepsy (Moritani et al., 2005), and this evidence supports our finding with regard to the increased RD in the bilateral uncinate fasciculus, bilateral inferior longitudinal fasciculus, right superior longitudinal fasciculus 1, left superior longitudinal fasciculus 2 and 3. In fact, most of the tracts with higher weights in the first principal component as listed in Table 2 have been reported to exhibit structural abnormalities in previous DTI studies on MTLE, suggesting their involvement in forming an epileptic network of seizure activity (Bernhardt et al., 2013; Diehl et al., 2008; Focke et al., 2008; Gross et al., 2006; Liu et al., 2012). Therefore, the proposed brain age prediction model provides a clinical indicator of the white matter degeneration of an individual with unilateral MTLE.

**Fig. 7:**
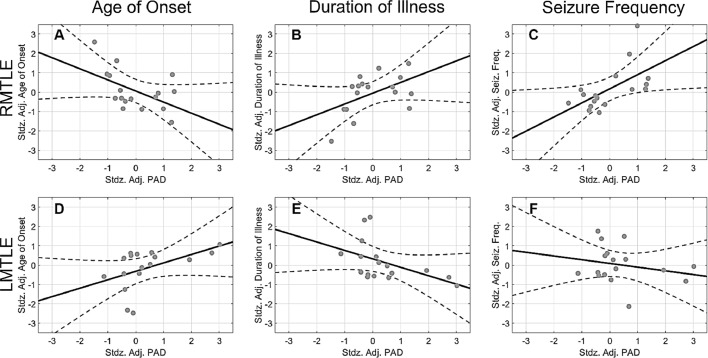
Association between clinical variables and predicted age difference (PAD) in patients with right or left mesial temporal lobe epilepsy. The solid dots, solid lines, and dashed curves in each plot indicate the observation, mean response, and 95% confidence interval of functions, respectively. In left MTLE a data point in the variable of “Age of Onset” exceeded 2.5 times the standard deviation, and was excluded from the correlation analysis. Horizontal and vertical axes denote standardized adjusted (denoted as “stdz. adj.”) PAD scores and clinical factors, respectively.

The increased PAD in patients with right MTLE, but not left MTLE, suggests that epileptic focus arising from the right hemisphere might distinctly affect brain structures, leading to more widespread white matter changes in the whole brain. This speculation is partially supported by the right hemi-aging model (Brown and Jaffe, 1975; Dolcos et al., 2002), which proposes that cognitive declines caused by physiological aging mainly influence those brain functions attributed to the right cerebral hemisphere rather than the left cerebral hemisphere. Evidence from a number of behavior studies supports this model, suggesting that right brain structures are more vulnerable to normal aging than the left ones (Dolcos et al., 2002; Goldstein and Shelly, 1981; McDowell et al., 1994). Liu et al. (2012) also found that, compared with major fiber tracts of the left hemisphere in left MTLE patients, right MTLE patients had more FA reductions in those tracts of the right hemisphere in concordance with our findings (Fig. 5). Hence, it is plausible that right MTLE causes more damages to the right hemisphere than left MTLE causes to the left hemisphere, and in turn leads to apparently older white matter brain age with pronounced increase in PAD.

### 4.2 Clinical relevance of increased white matter brain age in unilateral MTLE

When regressing out factors of age, sex, and number of AED classes, correlation analysis of the patients with right MTLE demonstrated three relationships between clinical variables and PAD. First, age of onset was negatively correlated with PAD; patients with earlier onset showed larger discrepancies between their brain age and chronological age. Structural MRI studies involving children with new-onset epilepsy have shown that refractory seizure is more likely to disturb the trajectory of white matter development than that of gray matter (Hermann et al., 2010; Hutchinson et al., 2010). Another longitudinal study demonstrated that increases in both the volume and the microstructural integrity of white matter continue to young adulthood, reaching a peak approximately at 30 years old (Lebel and Beaulieu, 2011). Because patients with right MTLE in this study had mean age of onset of 12.5 years old (Table 1), the epileptogenic process or refractory seizure might have damaged the myelin sheath and altered the trajectory of white matter development, leading to premature brain aging (Pardoe et al., 2017). Second, duration of illness was positively correlated with PAD, indicating that patients with longer disease duration had more prominent premature brain aging. The hippocampal atrophy and the extent of structural abnormalities beyond the mesial temporal lobe have been shown to be related to disease duration in MTLE (Bernasconi et al., 2005; Bonilha et al., 2006; Govindan et al., 2008). Therefore, premature aging of white matter along the disease course might contribute to overestimated brain age. Taken together, increased white matter brain age in right MTLE could be attributed to the aberrant trajectory of white matter development or the cumulative damages to the white matter tracts. However, we observed collinearity between age of onset and duration of illness in patients with right MTLE; therefore, the exact mechanism of increased PAD cannot be ascertained from the current correlation results. Third, seizure frequency was positively correlated with PAD, linking more white matter damages with higher seizure frequency in right MTLE patients. This result was in line with findings of previous structural MRI studies that seizure frequency was related to the progressive brain atrophy, which not only occurred in mesial temporal structures but also extended to other remote brain regions (Bernhardt et al., 2009; Coan et al., 2009). It suggests that recurrent seizure might lead to white matter alterations or network reorganization that exacerbates premature white matter aging in right MTLE.

By contrast, left MTLE showed opposite trends in correlations of PAD with age of onset and duration of illness. Although these trends would not survive in strict multiple comparison correction and are counterintuitive compared to the explanations in the right MTLE, these findings might imply that left MTLE and right MTLE possibly have distinct processes of pathophysiology and brain plasticity in response to the progression of disease. Our results indicate that left MTLE patients with earlier age of onset or with longer duration of illness had younger brain age. Also, the PAD in patients with left MTLE had no associations with seizure frequency, implying that the changes in PAD may not attribute to the recurrent seizure in left MTLE. We speculate that these findings may be interpreted by the concept of use-dependent plasticity (Kalisch et al., 2006), that the hemispheric asymmetry of some brain functions at the younger age becomes more balanced at the older age, thus one hemisphere may enhance the capacity to compensate dysfunctions of the other during aging process. However, this speculation should be verified by further studies with large sample size.

### 4.3 Comparison of present brain age prediction model with previous studies

Since the first study on neuroimaging-based age prediction which was conducted by Franke et al. (2010), many studies have developed brain age prediction models using different neuroimaging features or machine learning algorithms (Table 3). In Franke's study, they used the gray matter features derived from T1-weighted images of 547 healthy participants and estimated the brain age using relevance vector regression. Recently, Cole et al. (2017) used the raw data of T1-weighted images and secondary morphological features, such as cortical thickness, from approximately 2000 healthy participants to build a brain age prediction model using convolutional neural network approach. In contrast to the substantially large amount of training data used in their studies, our study established the prediction model with comparable performance through a relatively small data set. This implies that the microstructural changes during aging process as probed by diffusion MRI might be more sensitive than the morphological changes probed by T1-weight imaging.

**Table 3:** Comparison of our brain age prediction model with other models. The metrics listed here present the best performance of model reported in each study.

Study	Our study	Franke (2010)	Mwangi (2013)	Cole (2017)
Sample size				
(training data)	300	547	188	2001
Age range	18–92	19–86	4–85	18–90
Materials	Diffusion indices			
(DSI)	GM VBM	Diffusion indices		
(DTI)	T1W GM/WM volume and raw data			
Approach	GPR	RVR	RVR	GPR/CNN
Rho	0.95	0.94	0.90	0.96/0.96
RMSE	5.8 (years)	5.9 (years)	8.9 (years)	5.4/5.3 (years)
MAE	4.6 (years)	4.6 (years)	6.9 (years)	4.4/4.2 (years)

CNN: convolution neural network; DSI: diffusion spectrum imaging; DTI: diffusion tensor imaging; GM: gray matter; GPR: Gaussian process regression; MAE: mean absolute error; Rho: correlation between predicted and chronological age; RMSE: root mean square error; RVR: relevance vector regression; T1W: T1-weighted; VBM: voxel based morphometry; WM: white matter.

To date, few studies that used diffusion MRI data to build the brain age prediction model. Mwangi et al. (2013) used diffusion scalar indices derived from DTI to estimate brain age of healthy participants. The performance of the model in that study, however, was less satisfactory than that in other studies (Table 3). It might be due to the limited sample, diffusion reconstruction method, the modeling approach or other modeling factors. In the present study, we used DSI and ReMAP approach to capture comprehensive microstructural characteristics of white matter. We further used tract-based automatic analysis to produce tract-specific profiles of microstructural properties as our input features. These tract-specific features can provide the interpretability of the brain age prediction, allowing us to identify the tracts with most significant contribution to premature brain aging in right MTLE.

Brain age studies have demonstrated the capacity of the brain age to detect aberrant aging in those who have suffered from neurological and/or psychiatric problems. However, the brain age predicted from a new observation should be inferred with cautions. The common and empirical threshold employed the MAE of 5 years or below (Cole and Franke, 2017a) to appraise the prediction performance of a prediction model. The use of this threshold implies that, even in the cognitively healthy population, individual heterogeneity of white matter integrity would produce a normative distribution of brain age regardless of the sample size and modeling approach.

Given that the brain age model is built from a group of healthy participants, the predicted brain age might be subjected to bias when the model is applied to patients with MTLE. Cerebral atrophy and lesions caused by epilepsy may potentially affect image registration, leading to bias in brain age prediction. We have addressed this concern in our image registration process. Specifically, we employed the advanced diffusion registration algorithm (Hsu et al., 2012) and the two-step registration strategy (Chen et al., 2015) to minimize the registration bias due to inter-group difference in morphological changes. The procedure ensures that the variation of the brain age in the MTLE patients reflects the disease-related alterations in white matter integrity at the microscopic level, rather than the morphological changes at the macroscopic level.

### 4.4 Limitations

The age distribution of the training set was not uniform across lifespans. Middle-aged adults (40–60 years) accounted for 20.7% of the training datasets, much less than the proportion of the younger (<40 years: 49.0%) and senior (>60 years: 30.3%) adults. Nevertheless, we believe the estimation of parameters in the prediction model was accurate because the aging process is continuous throughout lifespan, and the GPR model was applied to a sufficiently large sample. Second, in the results of the prediction model, the PAD scores of the training set were correlated with chronological age, resulting in age-dependent bias for individual prediction across different ages. The reason for this estimated bias could be explained in part by age-related pathological changes in older individuals that were not necessarily present in younger ones (Franke et al., 2010; Mwangi et al., 2013). In the present study, we addressed this bias by recruiting an age-matched control group in the PAD comparison and regressing out chronological age in the correlation analysis. Third, the present study adopted cross-sectional study design that limited us to give conclusive explanations for findings with regard of associations between PAD and clinical factors, such as age of onset and duration of illness. It warrants a longitudinal follow-up study to explicitly dissociate epilepsy progression from aging effect, and is sensitive to detect cumulative white matter degeneration along the disease course in patients with unilateral MTLE in the future. A longitudinal study design is able to track PAD changes over the time to provide a measure of long-term trajectory and guide the decision of therapeutic strategies. Fourth, the relative small sample size per patient group may result in lack of statistical power. In fact, the recruitment of pure unilateral MTLE patients was challenging and time-consuming. Nevertheless, a larger sample size is recommended to evaluate the reproducibility of our results in the future.

Compared to the brain age models using structural MRI data such as BrainAGE method (Franke and Gaser, 2019), the model trained using the features derived from diffusion MRI has less generalizability due to the scanner-dependent spatial variability of the diffusion signal (Mirzaalian et al., 2016). The model established in this study cannot be directly applied to the data acquired from the other sites. To address this problem, we have developed a prototypical framework to generalize diffusion MRI-based brain age models using transfer learning techniques, and the feasibility has been demonstrated in our recent report (Chen and Tseng, 2019).

## 5 Conclusion

In the present study, we developed and applied a machine-learning based brain age prediction model to investigate patients with right and left MTLE. Patients with right MTLE exhibited premature brain aging than did patients with left MTLE, suggesting a more aggravated white matter alteration in right MTLE. The high contribution of the affected white matter tracts to premature brain aging and strong correlations of PAD with clinical variables including age of onset, disease duration, and seizure frequency unveiled the structural underpinning and clinical relevance of premature white matter aging in right MTLE. In conclusion, the white matter brain age is a potentially useful indicator of white matter alteration and disease severity in patients with right MTLE.

## Ethical approval

All procedures performed in this study involving human participants were in accordance with the ethical standards of the National Taiwan University Hospital (NTUH) Research Ethics Committee (REC) and with the 1964 Helsinki declaration and its later amendments or comparable ethical standards. Informed consent was obtained from all individual participants included in the study.

## Declaration of Competing Interest

The authors declare that they have no financial/non-financial and direct/potential conflict of interest.

## Appendix Supplementary materials

Image, application 1

Image, application 2
