# Supplementary material for: Premature white matter aging in patients with right mesial temporal lobe epilepsy: A machine learning approach based on diffusion MRI data
Source: Neuroimage Clin. 2019 Oct 23;24:102033. doi: 10.1016/j.nicl.2019.102033 (PMC6978225; doi:10.1016/j.nicl.2019.102033)
Supplement: Supplementary file 1 [file mmc1.docx]

**Supporting Information**

*S1 Magnetic resonance imaging database for the brain age prediction model*

To develop the brain age prediction model using diffusion MRI data, we used two data sets for model training and testing. The data set included images of T1-weighted (T1W) imaging and diffusion spectrum imaging (DSI) acquired from the same 3T MRI system (TIM Trio, Siemens, Erlangen) at National Taiwan University Hospital (NTUH). The training data set constituted 300 healthy individuals (age: 18–92 years, 44.89 ± 19.25 years; sex ratio: 123 male/177 female), and the test data set comprised 40 healthy individuals (age: 20–74 years, 44.58 ± 19.60 years; sex: 21 male/19 female). These individuals had no history of neurological or psychiatric disease. The data come from a collection of normal control groups from different research projects executed by the corresponding author of the present study. Each project was approved by the Institutional Review Board of NTUH. Informed consent was obtained from each participant.

*S2 Image quality assurance*

Before starting data analysis, DSI images were subjected to quality assurance procedures, including examination of the signal-to-noise ratio (SNR), number of motion-induced signal dropout, and degree of alignment between T1W and DSI images. SNR was evaluated by calculating the mean signal of an object divided by the standard deviation of the background noise (Dietrich et al., 2007). In practice, the signal was determined using a central square of an image with a size of 20 × 20 pixels, and the noise was averaged from four corner regions with a size of 10 × 10 pixels each. DSI images which had SNR within the mean SNR ± 2.5 standard deviations were included in the analysis. Because of relatively long scan time of DSI, in-scanner head motion would inevitably cause signal dropout, particularly in those with high *b* values. All acquired DSI images (5712 images per participant) were examined by comparing the signal in the central square of each image with the predicted signal attenuation. Signal deviation from the predicted distribution was considered signal loss. Data with less than 60 images of signal dropout per participant (1% of the total diffusion-weighted images) were included in the analysis. The degree of within-subject alignment between T1W and DSI images was evaluated by calculating the spatial correlation between the T1W-derived white matter tissue probability map and DSI-derived generalized fractional anisotropy map. Higher spatial correlation indicated better spatial alignment between T1W and DSI images. The datasets within the mean spatial correlation ± 2.5 standard deviations were included in the analysis. All data used in this study passed the quality assurance criteria.

*S3 Normative model*

Data in the normative model were retrieved from the same database mentioned in S1, comprising the data obtained from 524 normal individuals (age: 7–92 years, 33.17 ± 22.26 years; sex: 292 males/232 females). This cohort included the entire training data set for brain age modeling and another data set obtained from healthy children and adolescents. The normative model built from this data set across lifespan provided mean and standard deviation of each diffusion index for each tract at each year of age. A representative result is shown in Supporting Information Figure 1. The mean and standard deviation in the normative model were calculated as follow. First, all normal individuals in the database were stratified into male and female populations. Second, the estimated mean and standard deviation of each diffusion index for each tract were calculated at each year of age. Practically, the statistical parameters at a specific age were estimated by including the normal individuals whose ages were within the interval of the specific age ± 5 years. The calculated parameters across lifespan were then smoothed using cubic smoothing spline interpolation to reduce the variance from sampling bias. In this way, the normative model across lifespans was built to provide statistical parameters of each diffusion index for each tract, allowing one to quantify the normalized diffusion index against the estimated population on the individual basis.


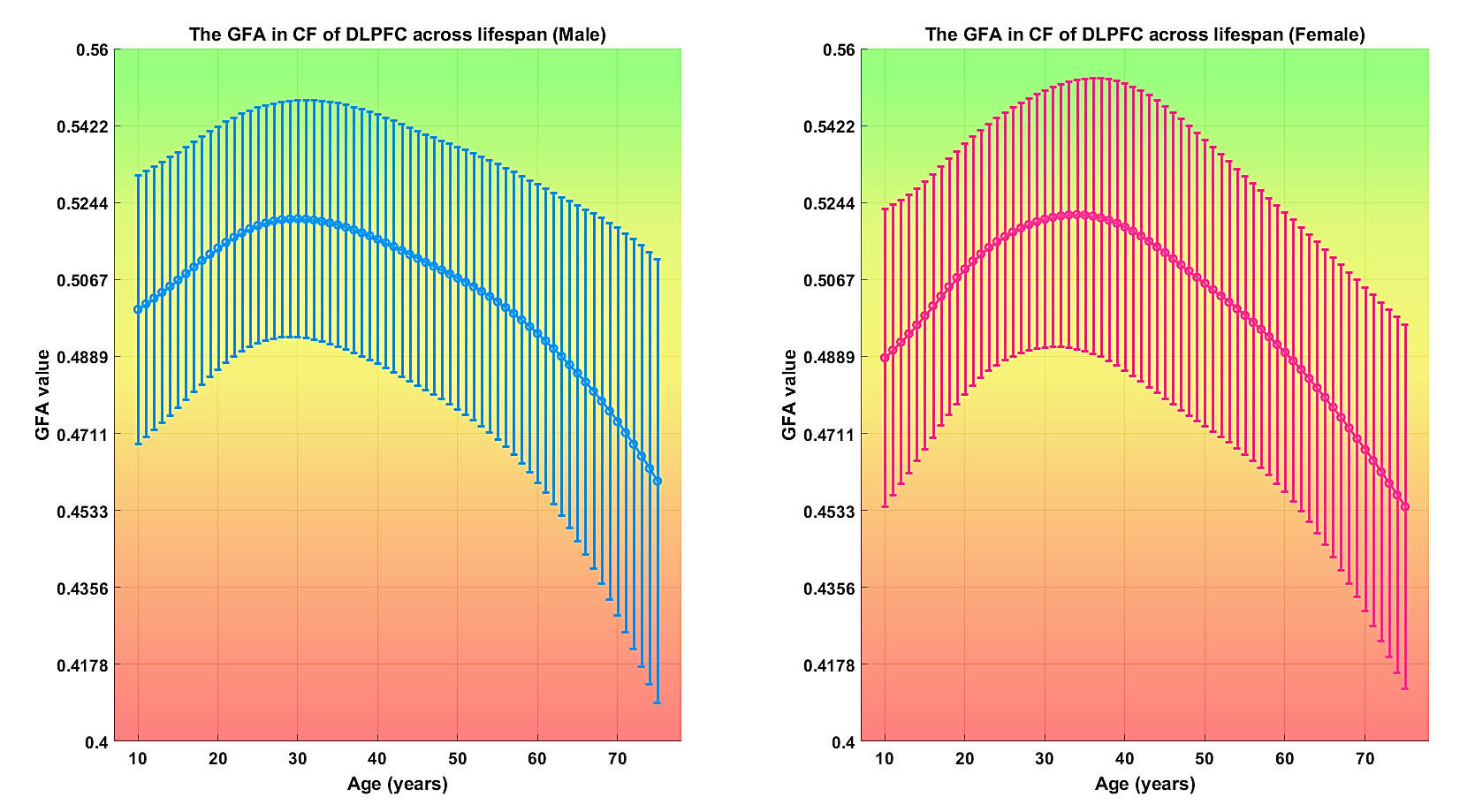


Supporting Information Figure 1: This figure demonstrates the normative model of the tract “Callosal Fibers connecting the Dorsal Lateral PreFrontal Cortex” (CF of DLPFC) stratified by sex. The normative model recorded the estimated mean and standard deviation of generalized fractional anisotropy (GFA) at each year of age. The lines in the figure represent the means of GFA at each year, and the bars represent ±1 standard deviations from the means.

*S4 Bootstrap analyses for correlation between clinical factors and brain age metrics*

To verify whether these associations were indeed false discoveries, we performed bootstrap simulations (5,000 times resampling from the data space) to construct the empirical distributions of correlation coefficients and estimated the confidence interval of the statistics. Supporting Information Figure 2 below shows the results of simulated empirical distributions. The associations between PAD and clinical metrics in RMTLE and LMTLE are shown in the upper and lower rows, respectively. According to the empirical confidence intervals of the correlation coefficients in the LMTLE group, the associations estimated by the original correlation analyses and bootstrap simulations are consistent and comparable (Supporting Information Table 1). Although we cannot rule out the possibility that the results might change if larger samples were used, the bootstrap analysis on the current data verified that RMTLE and LMTLE have two distinct patterns of correlations, suggesting that the correlations shown in the LMTLE group are not false discoveries.


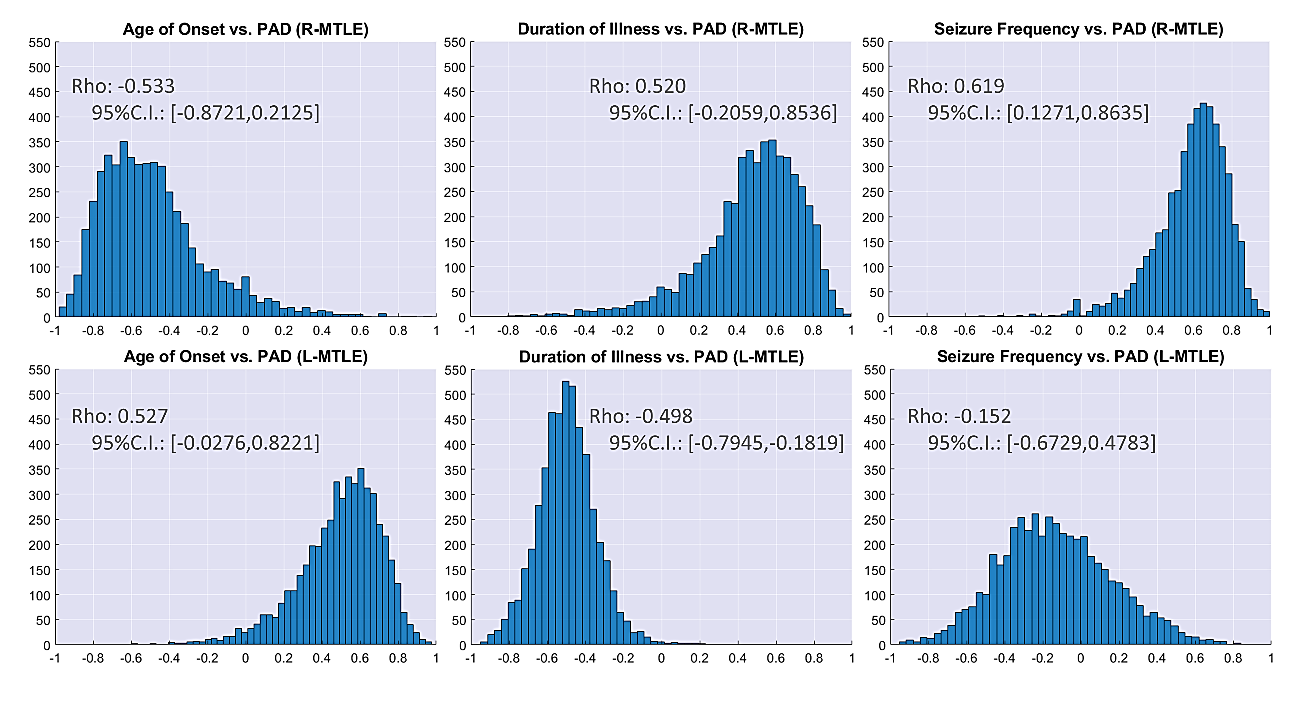


Supporting Information Figure 2: The empirical distributions of correlation coefficient derived from bootstrap simulation in RMTLE (upper row) and LMTLE (lower row).

|  | Age of onset | | Dura. of ill. | | Seiz. freq. | |
| --- | --- | --- | --- | --- | --- | --- |
|  | origin | bootstrap | origin | bootstrap | origin | bootstrap |
| RMTLE | -0.511 | -0.533 | 0.501 | 0.520 | 0.635 | 0.619 |
| LMTLE | 0.591 | 0.527 | -0.484 | -0.498 | -0.152 | -0.152 |

Supporting Information Table 1: The associations estimated by the original correlation analyses and bootstrap simulations.

References:

Dietrich, O., Raya, J.G., Reeder, S.B., Reiser, M.F., Schoenberg, S.O. (2007) Measurement of signal-to-noise ratios in MR images: influence of multichannel coils, parallel imaging, and reconstruction filters. J Magn Reson Imaging, 26:375-85.
