# Supplementary material for: Premature white matter aging in patients with right mesial temporal lobe epilepsy: A machine learning approach based on diffusion MRI data
Source: Neuroimage Clin. 2019 Oct 23;24:102033. doi: 10.1016/j.nicl.2019.102033 (PMC6978225; doi:10.1016/j.nicl.2019.102033)
Supplement: Supplementary file 2 [file mmc2.zip › Script/Description_about_files.docx]

This folder contains 4 files, namely “Connectogram_MTLE&Control.mat”, “PreloadMaterials_model&weights.mat“, “Script_Inference.m”, and ‘smo3D.m’.

The file “Connectogram_MTLE&Control.mat” includes the connectograms and the demographic data (age and sex) from patient and control groups.

The file “PreloadMaterials_model&weights.mat” includes the pretrained models (autoencoder and GPR model) and the statistical weights used in the data preprocessing.

The file “Script_Inference.m” is a script for brain age inference running in the MATLAB environment.

The file “smo3D.mat” is a function used in the script of brain age inference.
